# Quality of Work Life Amongst Nurse Professionals: A Concept Analysis

**DOI:** 10.3390/ijerph22111747

**Published:** 2025-11-19

**Authors:** Michelle Carneiro Fonseca, Vinícius Rodrigues de Oliveira, Samuel da Silva Guedes, Tallita Ormecinda do Espírito Santo Gomes, Debora Augusta Oliani Caravina, Katarine Florêncio de Medeiros, Dayara Ainne de Sousa Araújo, José Leonildo Fernandes de Queiroz, Richardson Augusto Rosendo da Silva, Jonas Sâmi Albuquerque de Oliveira, Quenia Camille Soares Martins

**Affiliations:** Departament of Nursing, Federal University of Rio Grande do Norte, Natal 59078-970, Brazil; michellecarneiro3112@hotmail.com (M.C.F.); samueldasilvaguedes@gmail.com (S.d.S.G.); tallita.gomes.706@ufrn.edu.br (T.O.d.E.S.G.); deboracaravina@hotmail.com (D.A.O.C.); katarinefmedeiros@gmail.com (K.F.d.M.); dayara-ainne@hotmail.com (D.A.d.S.A.); sidint01a@gmail.com (J.L.F.d.Q.); rirosendo@hotmail.com (R.A.R.d.S.); jonas.albuquerque@ufrn.br (J.S.A.d.O.); queniacamillesm@gmail.com (Q.C.S.M.)

**Keywords:** work, quality of life, occupational health, nurse

## Abstract

Exploring Quality of Work Life (QWL) is essential, and in the context of nursing, it becomes even more relevant given the significant contributions of nurses to patient health. This study aimed to define and clarify the concept of QWL among nurses through a conceptual analysis based on the model proposed by Walker and Avant. This is an Integrative Review (IR) conducted in accordance with the PRISMA protocol. Given the focus of this research, the adopted method was aligned with the steps proposed by Walker and Avant. The review included a sample of 14 articles, from which the use of the concept was identified as being centered around three major areas and seven key attributes: job satisfaction, recognition, adequate remuneration, job stability, favorable physical work environment, positive relationships with the team and patients, and autonomy in decision-making. In conclusion, QWL among nurses is defined as achieving a level of well-being in the workplace that allows nurses to feel safe, at peace, comfortable, and healthy—and to carry this state of well-being beyond the work environment.

## 1. Introduction

Discussions surrounding Quality of Work Life (QWL) gained greater emphasis beginning in the 1950s. During this period, an approach emerged that sought to understand both the social and technical aspects of work configuration through the reorganization of tasks, aiming to reduce suffering in workers’ lives and to propose improvements in working conditions [1].

Approximately two decades later, in the 1970s, the term QWL was coined by Louis Davis while developing a project focused on the conceptualization of job design. According to Davis, the concept of QWL refers to a concern for the overall well-being and health of workers during the performance of their professional activities [2].

QWL is closely linked to the occupations performed by individuals within the work environment, directly influencing their health, performance, behavior, satisfaction, and overall standard of living in society [3].

In Brazil, QWL is characterized by two main approaches: an assistance-based and a preventive approach. The assistance-based approach, which remains dominant, considers the construction and maintenance of well-being in the workplace to be the worker’s own responsibility. In contrast, the preventive approach advocates for a worker-centered perspective, aiming to reduce discomfort and promote well-being, with a primary focus on adapting the work environment rather than the individual [4].

Chiavenato states that QWL comprises various components, including satisfaction with the work performed; opportunities for professional growth within the organization; recognition for achievements; salary; benefits; interpersonal relationships within the team and institution; psychological and physical work environment; autonomy and responsibility in decision-making; and opportunities for participation in such decisions. Therefore, achieving QWL fundamentally entails satisfaction with workplace well-being, adequate compensation, and the ability to contribute to the work organization [2].

In this context, the importance of QWL in Nursing is particularly noteworthy, as it directly influences the outcomes of care provided by nursing teams to patients [5]. Although the concept of QWL is widely used in organizational management, the literature reveals inconsistencies, especially regarding its definition and application within the nursing context. This is primarily due to the fact that the term encompasses multiple components and may be interpreted in various ways. Such inconsistencies can hinder the effective implementation of QWL-related policies and programs aimed at improving the well-being of healthcare professionals.

It is also important to highlight the scarcity of scientific studies and public policies specifically focused on QWL in the field of Nursing, which underscores the conceptual gaps surrounding the topic [6,7]. Therefore, it is essential to address these gaps in order to foster both theoretical and practical advancements in the area. To this end, this study aims to define and clarify the concept of QWL among nurses through a conceptual analysis based on the model proposed by Walker and Avant.

## 2. Method

This study is an Integrative Review (IR) conducted in accordance with the PRISMA protocol [8]. Given the theme of this research, the method followed the steps described by Walker and Avant (2005) [9], which include the following phases: selection of the concept; establishment of the objectives of the analysis; identification of uses, attributes, model case, additional cases, antecedents, and consequences; and finally, definition of the empirical referents. Data collection was carried out during the months of October, November, and December 2023.

The Integrative Review (IR) is a strategy that reflects the researcher’s interest in synthesizing a range of studies on a particular topic, aiming to develop more comprehensive explanations on a specific subject [10].

The integrative review follows six phases, which are: (a) identification of the topic and formulation of the guiding research question; (b) establishment of inclusion and exclusion criteria for study selection; (c) data collection from the selected studies; (d) critical analysis of the selected studies; (e) interpretation of the results; and (f) presentation of a synthesized content-based review [11].

In alignment with the first phase, the following guiding question was formulated: “What is the concept of QWL among nurses as presented in the literature?”

To formulate the above-mentioned question, the PCC strategy (Population, Concept, Context) was used, as it is widely applied in scoping and integrative reviews [12]. It is important to highlight that the terms related to this strategy were adapted for use in each data source, considering variations and using the Boolean operators AND and OR to develop the final search strategies, as shown in Table 1.

The research was conducted using the following databases: Latin American and Caribbean Health Sciences Literature (LILACS), Medical Literature Analysis and Retrieval System Online (MEDLINE), Nursing Database (BDENF), and Scientific Electronic Library Online (SciELO). These data sources were selected due to their relevance within the field of Nursing.

The search strategy was tailored for each database, utilizing Health Sciences Descriptors (DeCS) and Medical Subject Headings (MeSH). These terms were combined using the Boolean operators “AND” and “OR,” as demonstrated in Table 2.

The following inclusion criteria were applied for the selection of studies: full-text articles available free of charge in English, Spanish, or Portuguese that adequately addressed the research question. Duplicate articles, as well as editorials and abstracts from scientific events, were excluded to ensure the quality and consistency of the analysis. The final selection of articles with the greatest thematic relevance is presented in Table 3.

## 3. Results

**Figure 1:**
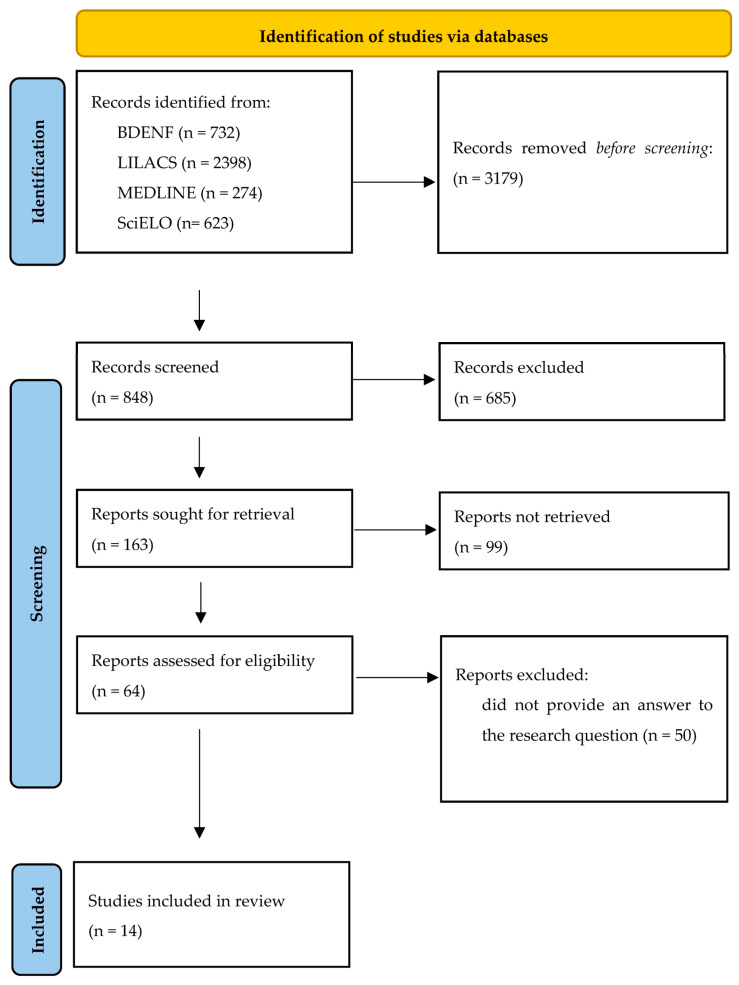
Study selection flowchart.

The initial search yielded 4027 studies. After applying the eligibility criteria, 14 articles were selected, as they provided answers to the central research question. The detailed selection process leading to the final sample is presented in Figure 1.

### 3.1. Presentation of the Studies Selected for the Review

Table 3 presents a synthesis of the studies selected for the sample, as well as the concept of quality of work life, aiming to associate it with the nursing context.

### 3.2. Uses and Defining Attributes of the Concept

Several relevant studies addressing the theme of quality of work life (QWL) among nurses were identified. These studies enabled the recognition of the various uses attributed to the concept, as well as its related attributes, providing a foundation for its critical analysis and refinement, as presented in Table 4.

Although there is no precise definition of the concept of QWL among nurses in the literature, it was possible to identify elements related to this concept in several studies. In these studies, the concept is represented through synonymous terms, and its use is concentrated in three core areas of nursing practice: care (78,5%), education (14.3%), and management (7.2%). Regarding the defining attributes, seven key elements were identified, which contribute to shaping the concept explored in this study.

### 3.3. Identification of the Model Case Related to the Concept

The model case represents the use of the concept through all of its defining attributes; in other words, it serves as a clear example of the concept. This case may be referenced from the literature, based on real-life experiences, or entirely fictional [9]. The following example was developed:

A nurse arrives at her workplace at 6:45 a.m. for the day shift, which officially begins at 7:00 a.m. In conversation with a colleague, she expresses great satisfaction with her work environment and mentions that, after completing her master’s degree, she received a significant incentive through the hospital’s Career and Salary Plan. She also notes that the management has been working to improve workplace conditions, citing positive changes such as the acquisition of ergonomic chairs, the implementation of scheduled breaks during the workday, and the resolution of shortages in Personal Protective Equipment. She considers her QWL to be above average.

### 3.4. Identification of Additional Case

To better delineate the concept, the creation of additional cases is recommended [9]. For this study, a contrary case was selected, that is, a situation presenting elements that oppose the QWL among nurses. The case is presented below: A nurse begins her night shift visibly exhausted after working consecutive overtime hours without adequate financial compensation. The physical work environment is inadequate: poor lighting, uncomfortable furniture, and unstable temperature conditions. During the shift, she shares with a colleague that she feels undervalued by the management team and frequently overwhelmed with tasks beyond her assigned responsibilities. She also faces difficulties in her relationships with some team members, marked by a lack of communication and collaboration. The nurse expresses insecurity in decision-making, stating that her autonomy is not respected. Furthermore, she reports feeling unmotivated, with no prospects for professional growth, and fears being laid off due to recent staff reductions. When asked about her Quality of Work Life, she states that she is extremely dissatisfied and is considering leaving the profession altogether.

### 3.5. Identification of Antecedents and Consequences

The most frequently cited antecedents in the literature refer to a welcoming and respectful work environment, as well as positive professional relationships. These elements underscore the importance of organizational processes in ensuring a satisfactory environment among team members, a fundamental condition for nurses to enjoy a good QWL. Regarding the identified consequents, they are related to improvements in work quality, increased productivity, and a reduction in employee illness.

It is important to highlight that antecedents correspond to events or conditions that predispose the occurrence of an outcome [27], in this case, factors that favor QWL among nurses. Conversely, consequents are phenomena that emerge from the presence of the concept [27]. Thus, an adequate QWL among nurses enables professionals to experience certain positive conditions and experiences within the work environment.

### 3.6. Identification of Empirical Referents

According to Walker and Avant (2005) [9], empirical referents consist of classes or categories of real phenomena that demonstrate the occurrence of the concept itself.

An employee at MCF Hospital reports that work processes and working hours are very well organized and that even interpersonal relationships among colleagues have improved. She mentioned an important health promotion activity being carried out, such as stretching exercises and yoga classes for employees, which brought her such satisfaction that she now goes to work every day with a sense of ease, as it has become a healthy place for her.

## 4. Discussion

QWL has become an essential focus for projects aimed at promoting the health and well-being of professionals [18,28]. In the context of healthcare work, especially in nursing, it is crucial to pay attention to these issues, considering the direct impact that these professionals have on planetary health, as they represent one of the largest workforces in the field [29].

The QWL constitutes a strategic factor in nurses’ management activities, as adequate working conditions and professional recognition increase engagement in team supervision, organization of care workflows, and implementation of protocols, thereby strengthening both the well-being of professionals and the quality of managerial and care processes [18].

When discussing QWL among nursing professionals, the centrality of this concept in care practices becomes evident, both in hospital settings and in primary health care. These contexts, due to their organizational characteristics and high care demands, prove to be conducive to work overload and, consequently, a reduced quality of life for these professionals [30].

In such a way, QWL constitutes a strategic axis both in management and in nursing care. In the managerial field, it involves ensuring adequate working conditions, material resources, and a collaborative environment. In care practice, it is reflected in safety and quality of care, since professionals with greater satisfaction and work–life balance tend to deliver safer and more patient-centered practices. Therefore, integrating QWL into management and care practices is essential to protect workers and enhance the quality of healthcare processes [15,30].

In the analysis of the attributes, job satisfaction stood out as the most relevant factor for ensuring QWL among nurses. This is because satisfaction depends on the presence of other essential factors, such as professional recognition and adequate compensation. In nursing, however, these elements are not yet fully consolidated: studies show that fair pay remains one of the main demands of these professionals. Combined with the instability of employment contracts, this reality leads many to assume exhausting workloads [15].

Thus, achieving an adequate QWL is influenced, among other factors, by salary-related aspects, including fair compensation that reflects the complexity of the tasks performed, and by organizational policies, which also contribute to workers’ satisfaction in these settings [15]. It is important to highlight that satisfaction and motivation are not limited to financial matters but also encompass respect and recognition for the role performed within the organization [31].

The physical conditions of the work environment are also essential attributes to ensure nurses’ health and well-being, as working in healthcare exposes professionals to various risks. In this regard, authors advocate for the continuous evaluation by institutions of the working conditions to which professionals are subjected, as improvements in this area can reduce absenteeism and adverse events in health services [16].

A positive work environment and good interpersonal relationships are other determining factors for consolidating QWL among nurses. This underscores the importance of promoting ongoing dialogue between supervisors and staff, offering workers opportunities to express their concerns, team-related issues, and other observed problems. Poor interpersonal relationships, especially among colleagues, can create tensions and result in negative consequences for the organization, contributing to stressful situations [32].

QWL is intrinsically linked to the promotion of continuous dialogue and health education in the workplace. Environments where communication is transparent and actively encouraged allow professionals to express their needs, concerns, and perceptions regarding care processes, thereby enhancing both team cohesion and patient safety. Simultaneously, the integration of health education strategies fosters the development of critical competencies, the updating of knowledge, and ethical reflection on professional practice—factors that increase job satisfaction and reinforce the quality of care provided. Therefore, investing in QWL through dialogue and education constitutes a decisive factor for the sustainability of care practices and the comprehensive well-being of healthcare professionals [30].

Building bonds is a challenging process, as newcomers need to establish credibility to build trust. Given the need for social integration and the development of new relationships, it is suggested that the concept of “social integration” be expanded to “intercultural social integration” in order to promote QWL for these professionals [33].

Employee participation in decision-making processes is also crucial, as it allows them to express their needs and identify vulnerabilities that need to be addressed. The well-being resulting from the achievement of these attributes allows the employer to anticipate the worker’s organizational commitment [34]. On the other hand, job dissatisfaction can cause problems for both the professional and the institution. The category of fair and adequate compensation includes salary-related matters and financial rewards, which should be compatible with the tasks performed, the workload, and market conditions [35].

Involuntarily, QWL affects personal life, since when there are professional conflicts or problems, it is often difficult for individuals to disconnect from them even after working hours. This leads to anxiety, persistent thoughts, attempts to find solutions, and other factors that result in emotional overload, ultimately harming both health and overall quality of life.

Therefore, to ensure quality of work life, it is essential to have a welcoming and respectful environment, along with positive professional relationships that reinforce the antecedents identified in this study. By consolidating QWL among nurses, it will be possible to achieve the outcomes identified herein, namely: improved work quality, increased productivity, and reduced illness related to professional activities.

QWL is built upon multiple factors encompassing both material and emotional or organizational aspects of work. Elements such as satisfaction with the work performed, professional recognition, adequate remuneration, and job stability constitute fundamental pillars for motivation and engagement. Additionally, the presence of a favorable physical environment, positive relationships with the team and patients, as well as autonomy in decision-making, enhances the perception of value and meaning in work. The integration of these elements promotes a balance between work demands and available resources, consolidating QWL and directly impacting the efficiency, safety, and quality of care provided in healthcare settings [31].

Hence, when implementing initiatives to improve quality of life, it is crucial to directly engage with the professionals involved, investigate their preferences, and develop measures tailored to the specific target population.

QWL transcends the workplace, directly impacting nurses’ quality of life outside the workplace. Adequate working conditions, professional recognition, autonomy, and healthy relationships promote physical and emotional well-being in other areas of life [34].

As a limitation of this research, it should be noted that many of the studies reviewed do not provide a clear definition of QWL. Most focus on related factors, workers’ opinions, and workplace relationships.

Therefore, it is recommended that further studies be conducted to clarify the concept of QWL, which is essential given that most professionals spend a large portion of their daily lives at work. It is critical to examine which elements are harmful and which are beneficial in these settings, in order to strengthen the latter and eliminate, or at least mitigate, the former.

## 5. Conclusions

The analysis of the concept of QWL among nurses is relevant as it fosters reflection on well-being in the workplace. Scientific evidence in this field can inform public health policies and be applied in contexts of work management and organization. The promotion of healthy behaviors in the professional environment, essential for the prevention of occupational diseases, benefits both institutions and workers.

In this context, the concept of QLW among nurses consists of achieving a level of well-being in the work environment that allows the nurse to feel safe, at peace, comfortable, and healthy, and able to enjoy this state even outside the work environment.

Accordingly, this study contributes to the advancement of scientific communication by addressing Quality of Work Life, taking into account the factors that may positively or negatively influence this domain.

## Figures and Tables

**Table 1:** DeCS and MeSH Descriptors/PCC Strategy.

	Descriptors	Decs	Mesh
P	Nursing professionals	Male and female nurses	Nurse Practitioners
		Nursing professional	Nurse
C	Quality of Work Life	Quality of Work Life	Quality of Life and Work
C	Work	Work environment	Working Environment
		Work	Work

**Table 2:** Search Strategy and Results.

Database	Search Strategy	Number of Results
LILACS	“Qualidade de vida no trabalho” OR “Quality of Life and work” OR “Nurse” OR “Qualidade de vida no trabalho” OR “Enfermeira or enfermeiro” AND “Qualidade de vida no trabalho” AND “Quality of Life and work” AND “Nurse” OR “Qualidade de vida no trabalho” AND “Enfermeira or enfermeiro”	419
BDENF	“Qualidade de vida no trabalho” OR “Quality of Life and work” OR “Nurse” OR “Qualidade de vida no trabalho” OR “Enfermeira or enfermeiro” AND “Qualidade de vida no trabalho” AND “Quality of Life and work” AND “Nurse” OR “Qualidade de vida no trabalho” AND “Enfermeira or enfermeiro”	369
MEDLINE	“Qualidade de vida no trabalho” OR “Quality of Life and work” OR “Nurse” OR “Qualidade de vida no trabalho” OR “Enfermeira or enfermeiro” AND “Qualidade de vida no trabalho” AND “Quality of Life and work” AND “Nurse” OR “Qualidade de vida no trabalho” AND “Enfermeira or enfermeiro”	37
SCIELO	“Qualidade de vida no trabalho” OR “Quality of Life and work” OR “Nurse” OR “Qualidade de vida no trabalho” OR “Enfermeira or enfermeiro” AND “Qualidade de vida no trabalho” AND “Quality of Life and work” AND “Nurse” OR “Qualidade de vida no trabalho” AND “Enfermeira or enfermeiro”	23

**Table 3:** Synthesis of Selected Studies Included in the Sample.

Articles	Authors	Methods	Results	Database	Concept of Quality of Work Life
Quality of Work Life of Nurses in a University Hospital [13]	Lima et al. (2013)	Cross-sectional descriptive study	Professionals who work satisfactorily can increase their productivity and professional quality, thus reflecting in the quality of care provided	LILACS	Quality of Work Life is an abstract and comprehensive concept, involving various aspects of human life such as satisfaction, professional recognition, social relationships, health, family, work, environment, among others
Quality of Work Life of University Nursing Faculty in Liquid Modernity [14]	Farias et al. (2023)	Descriptive study	The process of globalization and its social, economic, political, scientific, technological, and cultural transformations, especially impacting the labor market, is dominated by competitiveness and demands for competencies, skills, initiative, and clarity in work performance. Demotivation for work also results in flawed or insufficient execution of tasks, allowing the practice of presenteeism, which can be understood as “absenteeism” with physical presence	LILACS	Professional quality of life (PQL) consists of a combination of physical, technological, and socio-psychological factors capable of affecting the organizational climate and promoting worker well-being
Quality of Life and Job Satisfaction: Relationship Between Scales Evaluating the Constructs [15]	Rueda; Lima (2014)	Descriptive study	The results showed positive and significant correlations between the dimensions of the Quality of Work Life Scale and the factors of the EST, indicating that as Quality of Work Life increases, there is a proportional increase in job satisfaction	LILACS	The concept of Quality of Work Life refers to the need to humanize the work environment and to develop the human factor related to production processes, health, and the well-being of workers in their workplace, as well as other aspects of their lives that directly or indirectly influence how they perform their tasks. The construct is associated with aspects such as satisfaction, motivation, working conditions, and work management
Quality of Work Life: Discourse of Family Health Strategy Professionals [16]	Bracarense et al. (2015)	Descriptive study	The professionals attribute different meanings to the quality of work life, as well as recognizing the impact of this topic on improving user care	LILACS	The meanings attributed to Quality of Work Life by Family Health Strategy professionals encompass both subjective aspects, such as liking the profession and well-being at work, and factors related to working conditions and interpersonal relationships arising from work activities
Well-being at Work and Quality of Life: The Reality of the Hospital Nursing Team [17]	da Penha Silveira; Ribeiro; Mininel (2023)	Cross-sectional study	Nursing Quality of Work Life is directly related to the organizational support provided by institutions, which includes good practices such as goal setting, valuing ideas, autonomy, social support, modernization of technologies, and consequently, appreciation of work	BDENF	No formal concept was defined
Quality of Work Life: Evaluation of Intervention Studies [18]	Hipólito et al. (2017)	Integrative Review	It was found that there is a deficit of programs aimed at the health and well-being of workers, and that the implementation of effective policies in institutions could minimize this situation	SCIELO	It is a broad and committed understanding of living conditions in the work environment, including aspects of well-being, health, physical, mental, and social safety, and the capacity to perform tasks safely and with good use of personal energy
Sociodemographic and Environmental Influences of Work on the Quality of Life of Health Professionals [19]	Redu et al., (2024)	Descriptive-exploratory, cross-sectional, analytical, and quantitative study	A significant positive correlation was found between being a woman and the psychological domain and between income and the social and environmental domain while working hours were inversely related to general QoL. Additionally, workload negatively impacted the physical, psychological, and general QOL, furniture negatively influenced the psychological domain, and equipment was negatively associated with the physical and psychological domains.	SCIELO	No formal concept was defined
Quality of Work Life of Health Professionals in the Prison System [20]	Barbosa et al. (2018)	Cross-sectional descriptive study	The environmental/organizational aspect showed a low average satisfaction among health professionals, and this result may be related to the national prison environment, which is characterized by deterioration	SCIELO	No formal concept was defined
Workplace Violence and Professional Quality of Life Among Primary Care Nurses [21]	Fabri et al. (2022)	Cross-sectional study	It is necessary to develop institutional measures to promote professional quality of life, prevent workplace violence, and establish standard procedures to guide professionals in response to violent acts	SCIELO	No formal concept was defined
Quality of Work Life in Different Professional Areas in a Hospital [22]	Camargo et al. (2021)	Cross-sectional study	It was observed that in a hospital institution, workers in the care area have a lower Quality of Work Life compared to those in the administrative and medical areas. The administrative area also showed a lower Quality of Work Life than the medical area. Therefore, there is a need for inclusive workplace modifications to support workers who face greater difficulties	BDENF	No formal concept was defined
Quality of Life and Job Satisfaction: The Perception of Nursing Technicians Working in a Hospital Environment [23]	Renner et al. (2014)	Case study	It is necessary to reflect on the working conditions of nursing technicians, especially regarding the need to improve the quality of work life. There should be a reflection on the work environment and interpersonal and family relationships. It was observed that levels of job satisfaction significantly affect the lives of these professionals	BDENF	No formal concept was defined
Stressors in the Managerial Activity of Nurses: Implications for Health [24]	Silva et al. (2013)	Descriptive study	Stress resulting from the pace of life in the globalized world has led to the emergence of diseases that are closely linked to it. These conditions incapacitate the professional, causing harm to organizations, the worker, their family, and the community	BDENF	No formal concept was defined
Quality of work life and strategies to improve nursing education work [25]	Giordano et al. (2020)	Descriptive study	Work-related factors that influence Quality of Work Life (QWL) may include favorable human relations, a positive environment, well-being and satisfaction, and stress. Similarly, developing strategies to improve worker conditions highlights, firstly, that the institution must establish conditions to foster QWL through continuous improvements derived from institutional policies, contractual and collective systems, infrastructure, workplace climate, and work relationships. Secondly, it is up to the worker to implement individual strategies	BDENF	No formal concept was defined
Quality of Work Life of Primary Health Care Professionals [26]	Maganho et al. (2022)	Cross-sectional study	The analysis of the Quality of Work Life (QWL) of the Family Health Strategy team revealed that, according to the TQWL-42 questionnaire, professionals evaluated their QWL as unsatisfactory and neutral, yet they classified it as good. However, they emphasized that certain aspects could be improved	BDENF	The aim is to promote greater humanization of work and enhance professionals’ well-being, independent of the concept of job satisfaction. It is worth noting that this condition affects the health-disease process of professionals, their work environment, and personal life, reinforcing the need to reflect on Quality of Work Life

**Table 4:** Uses and Defining Attributes of the QWL Concept among Nurses.

**Uses of the Concept—QWL among Nurses**
Nursing Care
Nursing Education
Nursing Management
**Defining Attributes of the Concept—QWL among Nurses**
Satisfaction with the work performed
Recognition
Adequate remuneration
Job stability
Favorable physical work environment
Positive relationships with the team and patients
Autonomy in decision-making

## Data Availability

No new data were created or analyzed in this study. Data sharing is not applicable to this article.

## Abbreviations

The following abbreviations are used in this manuscript:

QWL	Quality of Work Life
WBW	Well-Being at Work
QoL	Quality of Life
WHO	World Health Organization
IR	Integrative Review
PCC	Problem, Concept, Context
CSP	Career and Salary Plan
PPE	Personal Protective Equipment
QL	Quality of Life
